# Characterization of tissue-specific differential DNA methylation suggests distinct modes of positive and negative gene expression regulation

**DOI:** 10.1186/s12864-015-1271-4

**Published:** 2015-02-05

**Authors:** Jun Wan, Verity F Oliver, Guohua Wang, Heng Zhu, Donald J Zack, Shannath L Merbs, Jiang Qian

**Affiliations:** Department of Ophthalmology, Wilmer Institute, Johns Hopkins University School of Medicine, Baltimore, MA USA; Department of Pharmacology and Molecular Science, Johns Hopkins University School of Medicine, Baltimore, MA USA; Department of Molecular Biology and Genetics, Johns Hopkins University School of Medicine, Baltimore, MA USA; Department of Neuroscience, Johns Hopkins University School of Medicine, Baltimore, MA USA; Institute of Genetic Medicine, Johns Hopkins University School of Medicine, Baltimore, MA USA; Institut de la Vision, Université Pierre et Marie Curie, 17 rue Moreau, Paris, France

**Keywords:** DNA methylation, Tissue-specific, Differentially methylated region, Gene regulation

## Abstract

**Background:**

DNA methylation plays an important role in regulating gene expression during many biological processes. However, the mechanism of DNA-methylation-dependent gene regulation is not fully understood. Here, we explore two possible DNA methylation regulatory mechanisms with opposite modes of gene expression regulation.

**Results:**

By comparing the genome-wide methylation and expression patterns in different tissues, we find that majority of tissue-specific differentially methylated regions (T-DMRs) are negatively correlated with expression of their associated genes (negative T-DMRs), consistent with the classical dogma that DNA methylation suppresses gene expression; however, a significant portion of T-DMRs are positively correlated with gene expression (positive T-DMRs). We observe that the positive T-DMRs have similar genomic location as negative T-DMRs, except that the positive T-DMRs are more enriched in the promoter regions. Both positive and negative T-DMRs are enriched in DNase I hypersensitivity sites (DHSs), suggesting that both are likely to be functional. The CpG sites of both positive and negative T-DMRs are also more evolutionarily conserved than the genomic background. Interestingly, the putative target genes of the positive T-DMR are enriched for negative regulators such as transcriptional repressors, suggesting a novel mode of indirect DNA methylation inhibition of expression through transcriptional repressors. Likewise, two distinct sets of DNA sequence motifs exist for positive and negative T-DMRs, suggesting that two distinct sets of transcription factors (TFs) are involved in positive and negative regulation mediated by DNA methylation.

**Conclusions:**

We find both negative and positive association between T-DMRs and gene expression, which implies the existence of two different mechanisms of DNA methylation-dependent gene regulation.

**Electronic supplementary material:**

The online version of this article (doi:10.1186/s12864-015-1271-4) contains supplementary material, which is available to authorized users.

## Background

DNA methylation regulates many biological processes, including development and disease by modulation of gene expression. Early studies on DNA methylation focused on CpG islands, DNA segments with a high density of CpG sites. Among the important findings about CpG islands are: (1) CpG islands tend to co-localize with the transcription start sites (TSS) of genes [1,2]; (2) promoter CpG islands are usually unmethylated (CpG island methylation is strongly associated with reduced gene expression) [1,2]; and 3) treatment of methylated CpG islands with methytransferase inhibitors generally increases gene expression [3]. CpG island methylation is also important for tissue-specific gene regulation. For example, certain tissue-specific genes are methylated in the tissues in which they are not expressed, but not in tissues where they are expressed [4,5]. However, the situation is more complex than a simple “on-off” model since the promoter CpG islands of some genes remain unmethylated even in cell types that do not express the gene [6].

It is generally accepted that DNA methylation represses gene expression. Recent technical advances, especially a variety of deep sequencing-based techniques, have made it possible to monitor DNA methylation patterns on a genome-wide scale [7-9]. Unbiased analysis of genome-wide methylation patterns reveals that DNA methylation is not always negatively correlated with gene expression. In fact, the methylation of a significant fraction of DNA methylation sites are positively correlated with gene expression [10], challenging the traditional view that DNA methylation represses gene expression. For the methylation sites that are positively correlated with gene expression, many questions remain to be answered. For example, are these methylation sites functional? Do they preferentially regulate downstream genes with certain gene functions? Do they interact with different TFs?

To address these questions, we utilized datasets of genome-wide tissue-specific DNA methylation and gene expression to perform a detailed survey of potential regulatory roles of tissue-specific differentially methylated sites (T-DMRs). The genome-wide methylation profiles were obtained by the improved comprehensive high-throughput array for relative methylation (CHARM) array [11], which has no bias to promoter and CpG islands and requires only small amount of tissue samples. We find that the T-DMRs that are positively correlated with gene expression are likely to be functional and that the genes associated with these T-DMRs are enriched for those that negatively regulate transcription and metabolism, suggesting a novel, two-layer mechanism of DNA methylation-dependent gene regulation. Furthermore, motif analysis reveals that distinct sets of TFs are likely to be involved in either positive or negative regulation mediated by DNA methylation. Huge difference in sequence composition between the two sets of motifs implies different regulatory mechanisms for DNA methylation-mediated gene regulation.

## Results

### A significant number of T-DMRs positively correlate with gene expression

**Figure 1:**
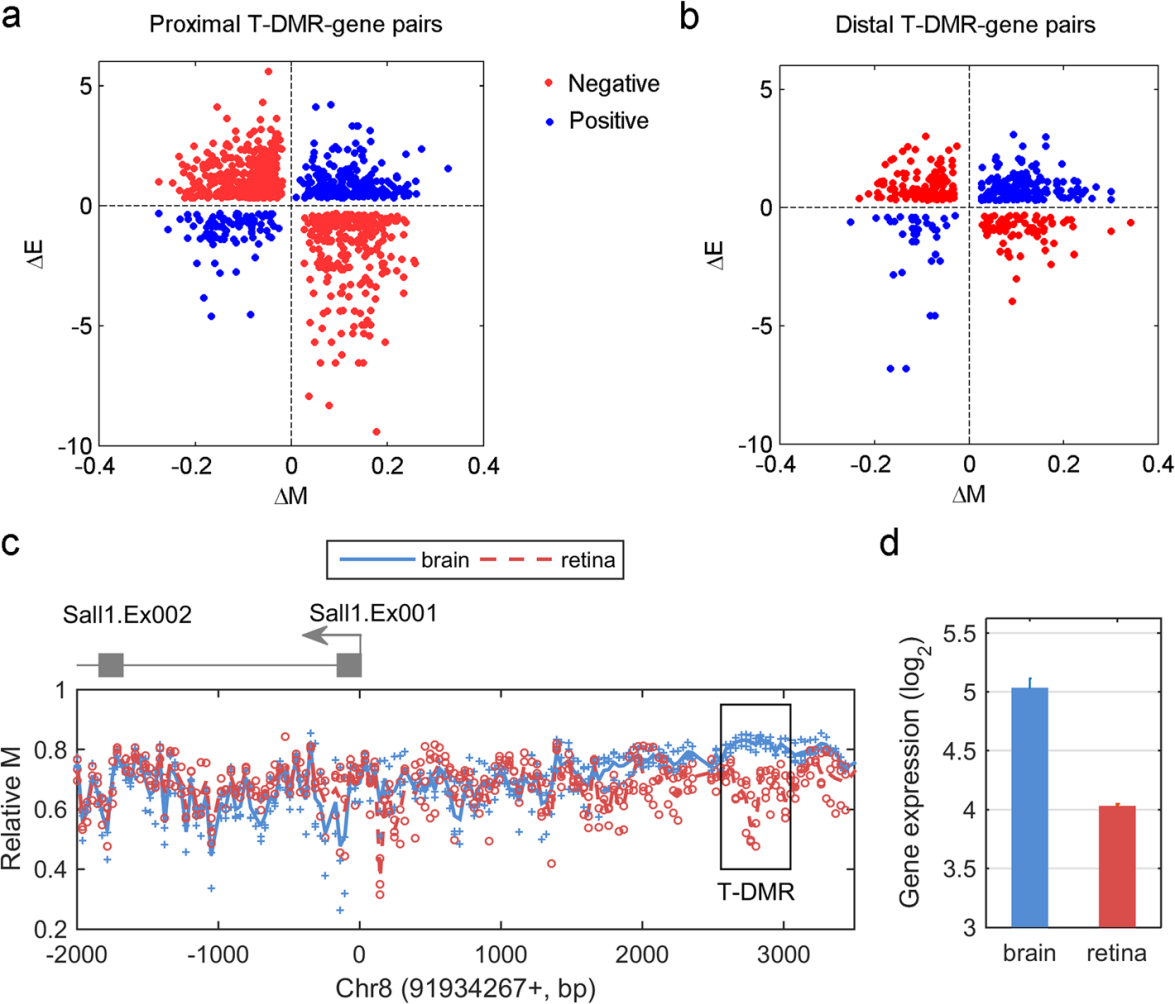
**A significant portion of differentially methylated regions show positive correlation with gene expression (blue dots). (a)** Scatter plot of ΔE and ΔM of proximal T-DMRs and genes. **(b)** Scatter plot of ΔE and ΔM of distal T-DMRs and genes. One example (gene *Sall1*) shows positive correlation between methylation difference within upstream T-DMR **(c)** and gene expression change **(d)**. The error bars in **(d)** represent the standard deviation of gene expression between triplicates of retina and duplicates of brain.

In our previous genome-wide profiling of DNA methylation profiles of mouse retina and brain [11], we identified 2498 T-DMRs. To explore the potential regulatory role of these T-DMRs, we integrated the methylation dataset with a genome-wide gene expression dataset obtained from the same tissues [12]. We compared the two tissues and calculated the difference in gene expression (ΔE) and the difference of DNA methylation (ΔM) of a T-DMR within a gene region (proximal T-DMR) from 4 kb upstream of the TSS to the end of transcription. We did not limit our interest to gene promoters since it has been shown that gene-body DNA methylation also plays a role in tissue-specific gene regulation [13]. In total, 952 unique proximal T-DMRs are associated with genes whose expression showed at least 25% fold change between the two tissues. The majority of the proximal T-DMRs (66%, red dots in Figure 1a) are negatively correlated with gene expression (negative T-DMRs), e.g. ΔM of the T-DMR and ΔE of the corresponding gene are in opposite directions. Methylation of the remaining 34% of T-DMRs (blue dots in Figure 1a) is positively correlated with gene expression (positive T-DMRs). This proportion is clearly different from a random simulation by shuffling actual ΔM and ΔE (49% and 51% for negative and positive T-DMRs, respectively).

In addition to the proximal T-DMRs, we also investigated distal T-DMRs located beyond 4 kb upstream of the TSS. To assign the potential target genes of these distal T-DMRs, we used the predicted enhancer-promoter relationships [14]. 297 unique distal T-DMRs (407 T-DMR-gene pairs) overlap enhancers, 49% of which are negative T-DMRs and 51% are positively T-DMRs (Figure 1b).

Figure 1c and d show one example of an upstream T-DMR positively that correlated with downstream gene expression. *Spalt-Like Transcription Factor 1* (*Sall1*) is a transcriptional repressor that plays a critical role in cortical neurogenesis [15,16]. We identified a T-DMR at about 3 kb upstream of TSS, where the methylation level of brain was higher than that of retina. In the expression microarray analysis, *Sall1* had a higher expression level (linear 2-fold change) in brain than in retina.

### Negative and positive T-DMRs are potentially functional

**Figure 2:**
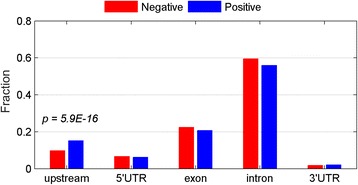
**Positive T-DMRs have similar genome locations as negative T-DMRs.**

To evaluate whether T-DMRs, especially the positive T-DMRs, might play a role in gene regulation, we analysed a series of genomic features of the positive T-DMRs and compared them with those of negative T-DMRs. In general, positive and negative T-DMRs have similar distributions at all genomic locations (Figure 2). Interestingly, 15% of positive T-DMRs are located upstream, compared to 10% of negative T-DMRs (p = 5.9 × 10^−16^, binominal model).

**Figure 3:**
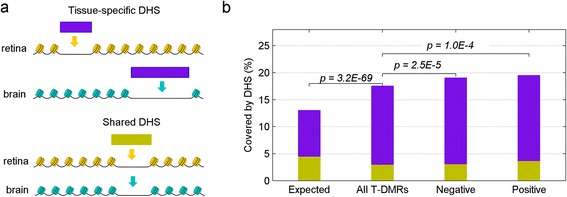
**T-DMRs are enriched in DHS regions. (a)** Schematic plot for tissue-specific DHS and shared DHS; **(b)** Percentage of T-DMRs covered by DHS.

Next, we compared the position of T-DMRs with known functional elements. Specifically, we examined the overlap of the T-DMRs and DNase I hypersensitivity sites (DHSs), short regions of open chromatin indicative of active transcription. Of the DHS regions observed in adult mouse retina and/or brain [17-19], 65% are present exclusively in retina or brain (tissue-specific DHSs) (Figure 3a). The remaining 35% are present in both tissues (shared DHSs). Interestingly, about 18% of T-DMRs are within DHSs, which is a significant overrepresentation compared with random expectation (13%, *p* = 3.2 × 10^−69^, Figure 3b), consistent with other reports [20]. More importantly, a large majority (83%) of T-DMRs within DHSs are located at tissue-specific DHSs. In contrast, only 17% of the T-DMRs overlap shared DHSs, which is significantly underrepresented compared to random expectation (35%, p = 1.3 × 10^−108^). These results suggest that T-DMRs are likely to play a role in tissue-specific gene regulation. Interestingly, a significant portion of both positive and negative T-DMRs are located within DHSs compared to that of all T-DMRs (Figure 3b, p = 2.5 × 10^−5^ and 1.0 × 10^−4^, respectively).

**Figure 4:**
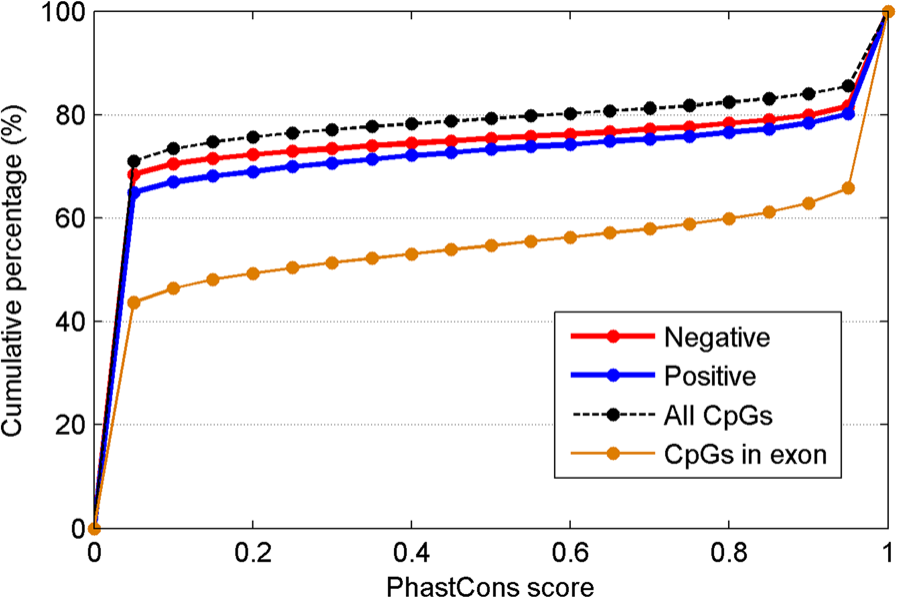
**Both positive and negative T-DMRs are more conserved than genomic background.** Cumulative distribution is shown as the percentage of CpG sites under the corresponding PhastCons score in negative T-DMRs (red), positive T-DMRs (blue), all probes on the CHARM array (black, as negative control), and exons (brown, as positive control), respectively. The distribution describes the percentage of the CpG sites that have a conservation score less than or equal to a given conservation score (x-axis). Basically, the curves on the top are less conserved than those on the bottom.

Sequences within T-DMRs are known to be more evolutionary conserved than other genome regions [21]. Here we examined the evolutionary conservation (PhastCons) of the CpG sites within negative and positive T-DMRs, and compared them to the CpG sites within the whole genome and in all exons on the CHARM array. Conservation scores of both negative and positive T-DMRs are higher than that of genomic CpG sites, but lower than those of CpG sites in exons only (Figure 4). Moreover, positive T-DMRs have more conserved CpG sites than negative T-DMRs.

In summary, our result suggests that positive T-DMRs are likely to play a functional role in regulating tissue-associated gene expression as indicated by several lines of evidence, including genomic location, overlap with functional elements and evolutionary conservation.

### Multiple T-DMRs can be associated with one gene

**Figure 5:**
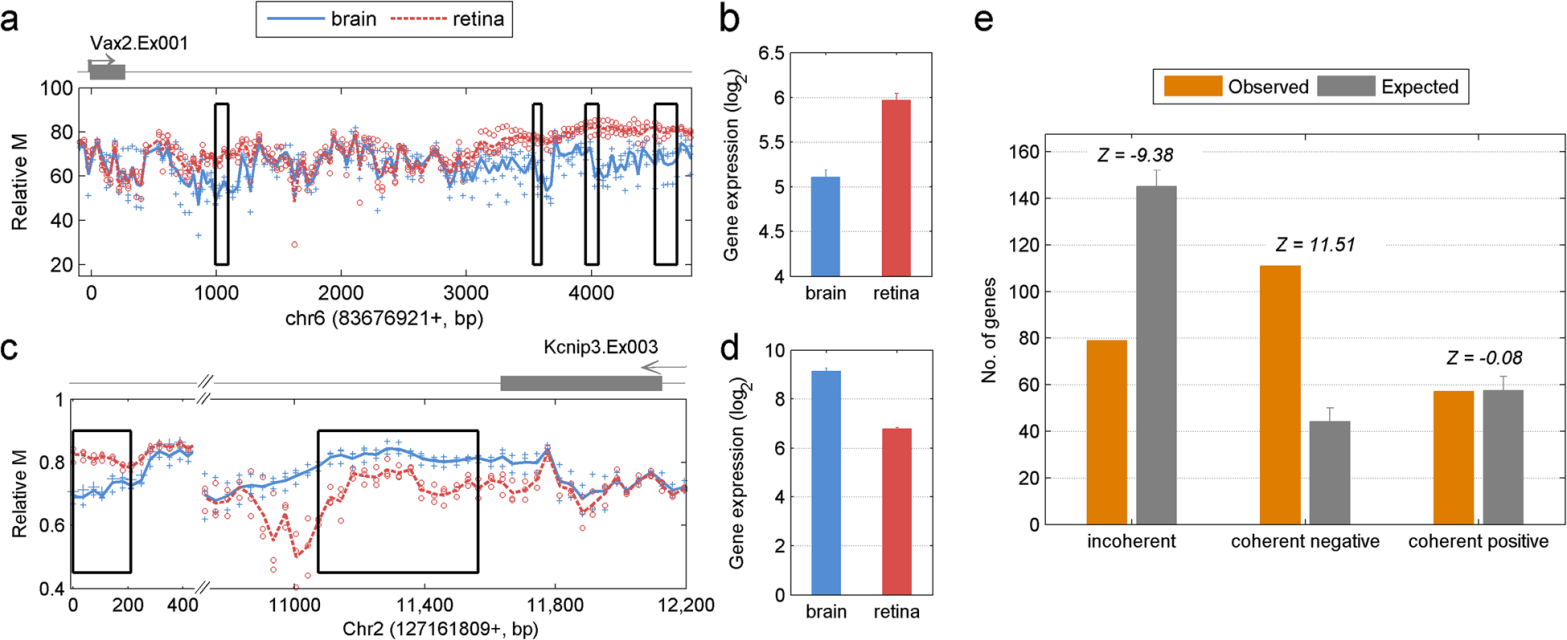
**Genes possibly regulated by multiple T-DMRs. (a)** 4 coherent T-DMRs found on *Vax2*; **(b)** Gene expression of *Vax2*; **(c)** 2 incoherent T-DMRs found on *Kcnip3*; **(d)** Gene expression of *Kcnip3*; **(e)** Number of genes with incoherent, coherent negative, and coherent positive T-DMRs, respectively. The error bars represent the standard deviation of randomly shuffling results for 10,000 times.

For genes that may be controlled by multiple T-DMRs, we divided T-DMR sets into coherent and incoherent, based upon whether or not the multiple T-DMRs had the same correlation direction with gene expression. For example, a coherent set of four positive DMRs were identified in the first intron of *ventral anterior homeobox 2* (*Vax2*), which plays an important role in retinal development [22]. The methylation level of these 4 T-DMRs are all higher in retina than in brain (Figure 5a), and gene expression of *Vax2* is almost linear 2-fold greater in retina than in brain (Figure 5b). In contrast, *Kv channel interacting protein 3, calsenilin (Kcnip3)* has an incoherent set of 2 T-DMRs in the third intron (Figure 5c) with one positive and one negative T-DMR. The gene expression data showed that *Kcnip3* had a linear 4-fold higher gene expression level in brain (Figure 5d).

Overall, 247 unique genes are associated with multiple T-DMRs. Among them, 79 are associated with incoherent T-DMR sets (e.g. *Kcnip3*). While keeping the same ΔE for each gene, after randomly shuffling the T-DMRs ΔM across the genes, we found that the expected number of genes associated with incoherent T-DMR sets was 145 (Figure 5e), suggesting that genes are less likely to be regulated by incoherent T-DMR sets (Z = −9.38). Of the 168 genes associated with coherent T-DMR sets, the expression of 111 genes is negatively correlated with the corresponding T-DMR set, which is much higher than expected (44 genes, Z = 11.51). In contrast, the number of genes associated with coherent positive T-DMRs (e.g. *Vax2*) is 57, the same as the expected distribution.

### Transcription repressors are more likely to be regulated by positive T-DMRs

**Figure 6:**
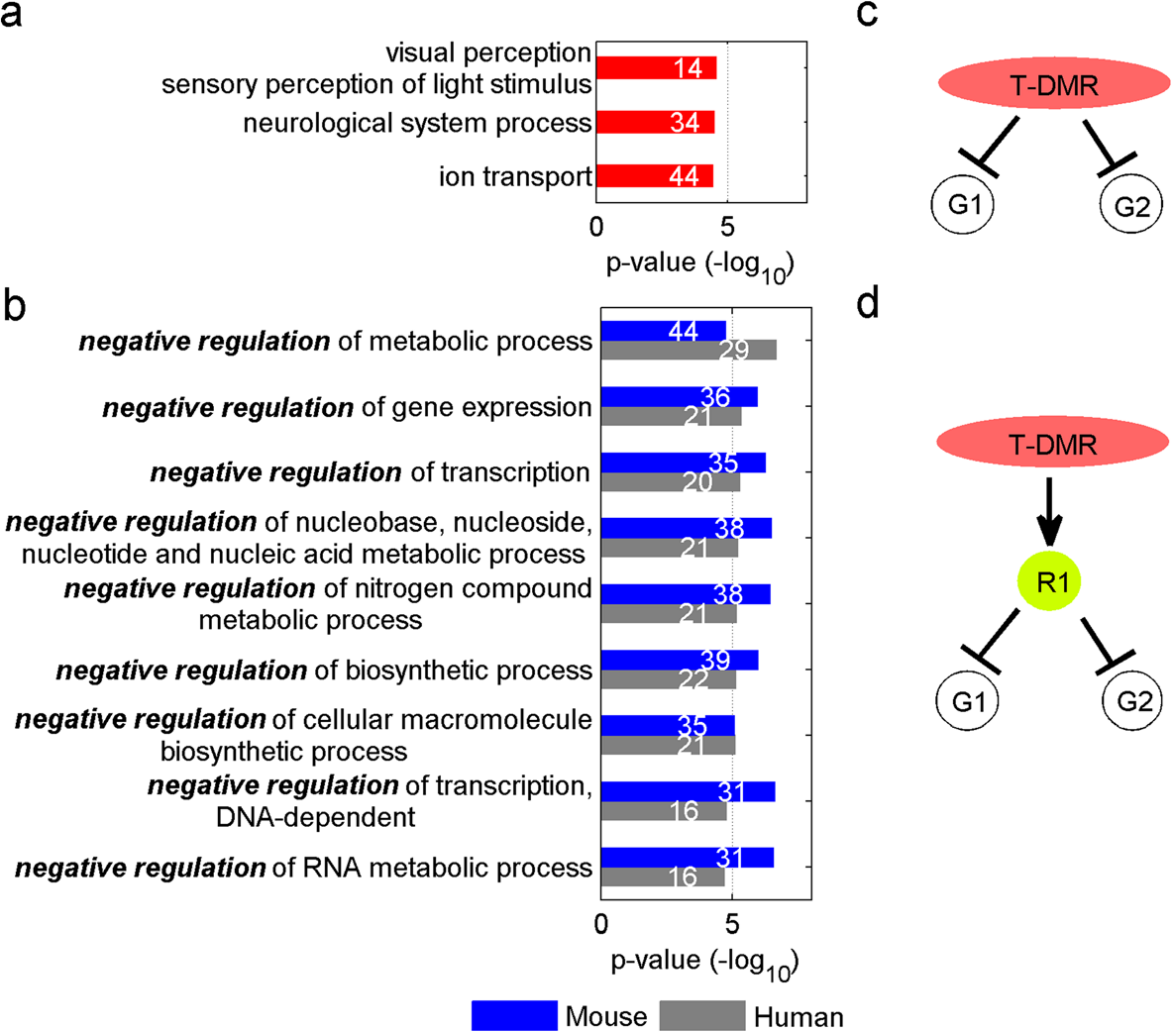
**Genes encoding transcriptional repressors are more likely to be associated with positive T-DMRs. (a)** GO functions that are enriched in genes correlated with negative T-DMRs; **(b)** GO functions that are enriched in genes correlated with positive T-DMRs for both mouse tissue data (blue) and human tissue data (gray); **(c)** and **(d)** Schematic plots of a T-DMR negatively and positively regulating gene expression, respectively. “R1” in **(d)** represents transcription repressors.

We performed gene ontology (GO) analysis on genes associated with either negative (534 genes) or positive (330 genes) T-DMRs. Genes associated with incoherent T-DMR sets were excluded for this analysis. It is not surprising to see that genes associated with negative T-DMRs are enriched for diverse types of biological functions such as “visual perception/sensory perception of light stimulus,” “neurological system process,” and “ion transport” (Figure 6a), reflecting tissue-specific features. Intriguingly, we found that genes associated with positive T-DMRs, e.g. *Sall1* and *Vax2* previously mentioned, often encode negative regulators such as “negative regulation of RNA metabolic process/nitrogen compound metabolic process,” “negative regulation of gene expression/transcription/transcription” and “negative regulation of biosynthetic process” (Figure 6b). In total, 74 genes out 330 genes (22%) whose expression was positively correlated with T-DMRs encoded negative regulators (compared to 15% in background, p = 4.3 × 10^−5^).

To test whether our finding could be generalized to other systems, we performed similar analysis on the data from a human tissue-specific DNA methylation study using brain and liver in an independent study published by Irizarry et al. [10]. In this study, 1023 and 175 genes were identified to be associated with a negative or positive T-DMR, respectively. Interestingly, the 175 genes associated with positive T-DMRs are also enriched for negative regulators (gray bars in Figure 6b).

Our finding suggests a new mode of DNA methylation-dependent regulation of tissue-specific expression: while the majority of genes appear to be directly inhibited by DNA methylation (Figure 6c), some genes may be indirectly inhibited by DNA methylation that is associated with expression of specific repressors (Figure 6d).

### Distinct sets of DNA motifs are associated with positive and negative gene regulation

To further explore the molecular mechanisms that differentiate possible positive and negative regulation via DNA methylation, we predicted the transcription factor binding sites that associate with the two types of T-DMRs. All possible 6-mers in the T-DMRs were enumerated and compared to the occurrence of each motif in randomly selected genomic regions. The significant motifs were identified based on p-values (see Methods).

**Figure 7:**
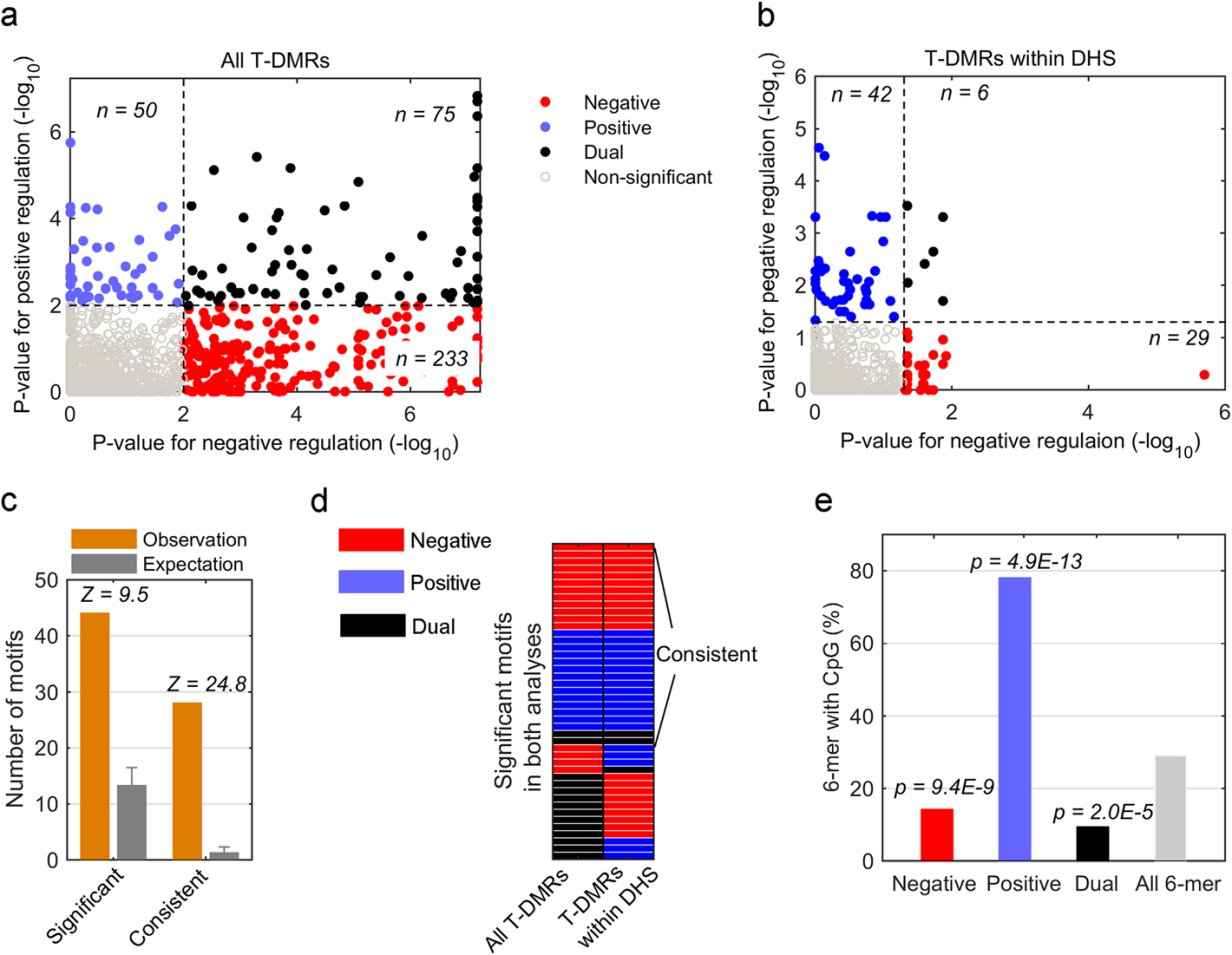
**Distinct sets of DNA motifs were found in positive and negative T-DMRs. (a)** 233 negative (red), 50 positive (blue), and 75 dual (black) found in all T-DMRs regulating gene expression for FDR < 0.01. Gray dots are other non-significant 6-mers in the study; **(b)** Similar results obtained in T-DMRs overlapped with DHSs for FDR < 0.05; **(c)** The numbers of significant motifs in both **(a)** and **(b)** and motifs playing the same regulation roles compared to expected numbers; **(d)** Heatmap of regulation role of significant motifs in both analyses; **(e)** Percentage of 6-mer motifs containing a CpG for each motif group in **(a)**. Gray bar is percentage of all 6-mer motifs with a CpG.

We predicted 233 and 50 motifs that are specifically associated with either negative or positive T-DMRs, respectively. An additional 75 motifs are associated with both positive and negative T-DMRs, which we termed “dual” motifs. The results suggest that positive and negative regulations are largely governed by distinct TFs (Figure 7a). If we focused on the T-DMRs that overlapped with DHSs only, 29, 42 and 6 motifs were obtained for negative, positive and dual regulation, respectively, (Figure 7b). Additional file 1: Table S1 lists all significantly enriched motifs identified in Figure 7a and b with corresponding p-values after FDR correction. The motif analyses in all T-DMRs and those within only DHS showed great consistency. First, 44 motifs were significant in both analyses, while only 13 motifs were expected (Z = 9.5) (Figure 7c). Second, among these 44 motifs, 28 were predicted to have the same regulation role (i.e. positive, negative and dual), while only 1 motif was expected to share the same role (Z = 24.8) (Figure 7c and d). Third, the enrichment score of all 6-mer occurences in two analyses also showed significant correlation (Additional file 2: Figure S1). The comparison suggested that our motif analysis was robust and not sensitive to the input set of T-DMRs.

Notably, those motifs that are associated with negative or positive T-DMRs have different numbers of CpG sites within the motif (Figure 7e). Only 14.2% of motifs associated with negative T-DMRs contain at least one CpG site. Similarly, 9.3% of the motifs associated with dual regulation contain a CpG site. Both of these were significantly depleted (p = 9.4 × 10^−9^ and 2.0 × 10^−5^, respectively), as 29.0% of all possible 6-mers contained a CpG. In contrast, 78.0% of motifs associated with positive regulation contain a CpG site, which is significantly enriched (*p* = 4.9 × 10^−13^). Our finding implies that the methylation might occur in the nearby CpG sites for the negative T-DMRs, while the methylation in the positive T-DMRs is likely to occur directly on the TF binding sites, suggesting again that there exist different regulatory mechanisms for positive and negative T-DMRs.

**Figure 8:**
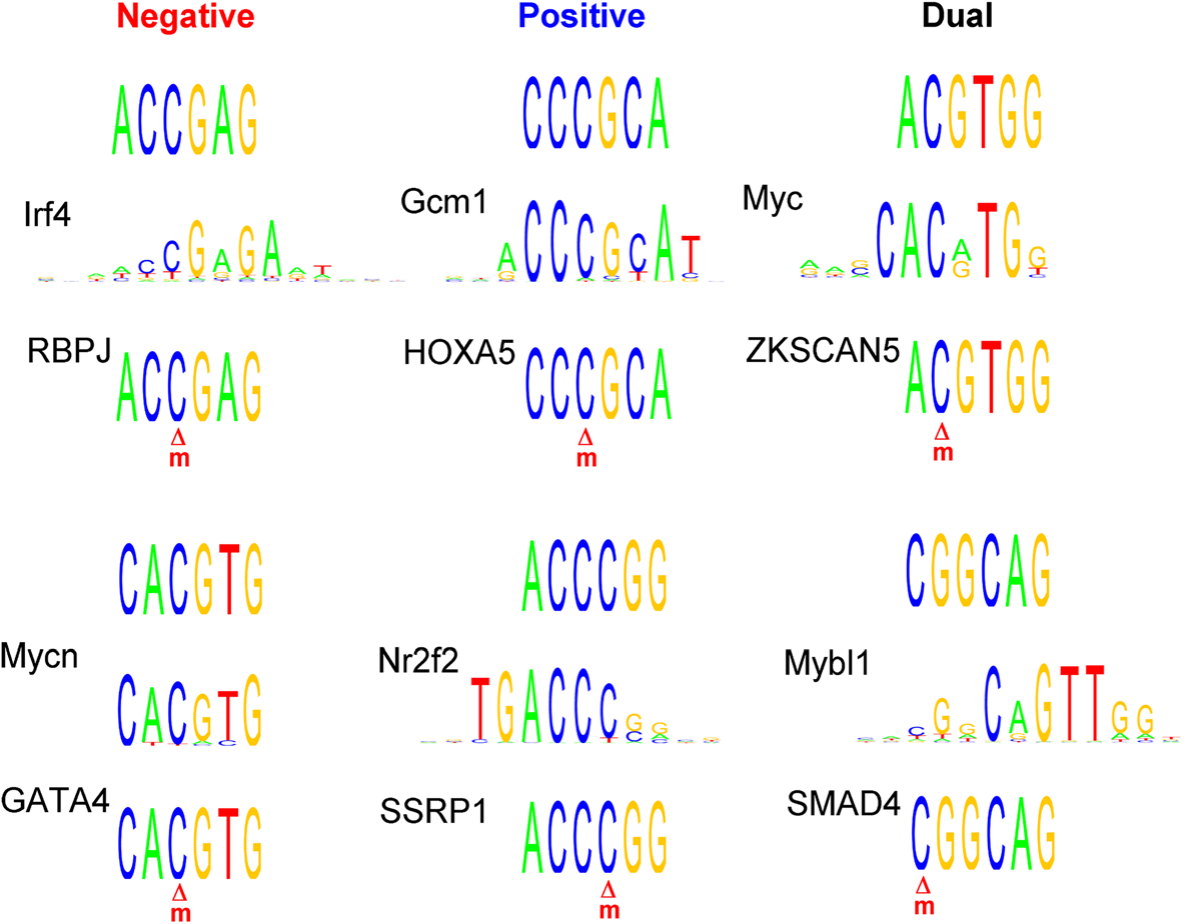
**Selected 6-mer motifs from negative, positive or dual T-DMRs containing CpG sites (top), similar binding sites of matched transcription factors from databases JASPAR and UniPROBE (middle), and human transcription factors known to bind in a methylation-dependent manner (21) (bottom).** The red “m” under the CpG represents methylated CpG binding site.

A recent study suggests that some TFs can recognize both methylated and unmethylated motifs [23]. Among the 358 motifs in the negative, positive and dual T-DMRs, 79 of them contain at least one CpG. We compared these 79 motifs to both known methylated and unmethylated TF-binding consensus sequences. Motifs were compared to unmethylated consensus sequences in databases JASPAR [24,25] and UniPROBE [26] using TOMTOM [27]. Additionally, motifs were compared to motifs identified in our previous work that preferentially bind to methylated DNA [23]. For each CpG-containing DNA motif, we obtained two TFs, one that recognizes the motif in the unmethylated state and the other in the methylated state. Figure 8 shows several examples for which a consensus sequence is predicted to bind different TFs based on the methylation state of the CpG within the motif. For instance, the motif ACCGCA from the T-DMR negative regulation group is similar to the binding sequences of *interferon regulatory factor 4* (*Irf4*). However, the methylated form of the motif can be recognized by *recombination signal binding protein for immunoglobulin kappa J region* (*RBPJ*) (Figure 8).

## Discussions and conclusions

CpG methylation has been thought to disrupt TF-DNA interactions either directly [28], or indirectly by recruiting sequence-independent methyl CpG binding domain proteins that occupy the methylated promoters and compete for the TF binding sites [29]. However, this traditional view has been challenged by several recent studies. For example, a mass spectrometry-based screen and a protein-binding microarray approach were recently used to identify TFs that preferentially bind to methylated CpG sites [23,30,31]. These results suggest that the mechanism of DNA methylation mediated gene regulation is more complicated than a purely inhibitory role.

In this study, we characterized T-DMRs and correlated them with the expression levels of associated genes. Methylation of the majority of T-DMRs negatively correlates with gene expression levels, consistent with the current notion of an inhibitory role for DNA methylation; however, the methylation of a significant fraction of T-DMRs is positively correlated with expression level. Positive T-DMRs are found more frequent in promoter regions than negative T-DMRs, as well as in DHS regions. Moreover, the positive T-DMRs are more conserved than negative T-DMRs. These results together suggest that the observed positive correlation is not simply due to chance and these positive T-DMRs are likely to play a regulatory role.

Although it has been shown by some groups that DNA methylation in gene bodies correlates with transcriptional activity [7], our observation is novel, and different to the previous findings. First, previous work [7] showed that non-CpG methylation (i.e. CHG and CHH methylation) within gene body is positively correlated with gene expression, while CpG methylation showed no correlation with gene activity. In our study, we focused on CpG methylation. Second, the published studies correlated DNA methylation and gene expression ranks among all different genes in one cell condition (e.g. stem cell line), while our study correlated the methylation difference and gene expression difference of the same gene between two tissues [7]. Third, the distributions of positive and negative DMRs throughout various genomic features (e.g. promoter, exon and intron) are very similar (Figure 2a). There is no enrichment for positive DMRs in gene body. Fourth, it has been found that CpG density is one major determinant for functionality of DNA methylation [5]. Davies et al. also observed that a significant overrepresentation of T-DMRs was located in low CpG density promoters [32]. Interestingly, in our study, the CpG ratios of positive T-DMRs are higher than that of negative ones (Additional file 2: Figure S2), suggesting that the positive T-DMRs are a novel discovery.

We find that genes whose expression negatively correlated T-DMRs are enriched for functions carried out in adult tissues, while the positively correlated genes were enriched for negative regulators such as transcriptional repressors. This possible two-layer regulation mechanism by positive T-DMRs may be unique to development and the establishment of tissue-specific expression. We do not observe a two-layer regulation when we analysed the association of cancer-specific DMRs (C-DMRs) and gene expression differences between colon cancer and normal tissue (data not shown) [10]. In the cancer study, genes whose expression is positively correlated with C-DMRs are not enriched for transcriptional repressors, suggesting that such a two-layer regulation mechanism is not an important feature in the development of cancer.

We find two distinct sets of motifs that associate with either positive or negative regulation of gene expression. While only 14% of the predicted motifs associated with negative gene regulation contain a CpG site, strikingly, 78% of the positive gene regulation motifs contained at least one CpG, suggesting that distinct sets of TFs participate in the different potential mechanisms of methylation-dependent gene regulation. For the positively associated motifs that contain a CpG site, it may be the methylation of that specific CpG, which allows the binding of a particular TF that only binds to methylated DNA and promotes transcription. Conversely, for negative T-DMRs, generalized methylation of the T-DMR may be more likely to inhibit transcription by the binding of methyl-binding proteins rather than a specific TF that only binds to methylated DNA. Clearly, we still have much to learn about the varied mechanisms by which DNA methylation can contribute to the regulation of gene expression, particularly in the establishment of tissue-specific expression during development.

As discussed previously, early analyses on DNA methylation often focused on promoter regions. However, accumulated evidence suggests that enhancers could activate gene expression independent of their distance to the promoters of target genes [33-36]. The enhancers could locate in distal intergenic regions and introns. For this reason, in our analysis, we did not limit T-DMRs to the promoter region. Nevertheless, if we performed the similar analysis on promoter regions, most of the results remain. For example, the enrichment scores for motifs from all T-DMRs and promoter-only analyses are significantly correlated (Additional file 2: Figure S3). In addition, we also performed the analysis on functional genes. 13 out of 67 (19.4%) genes positively regulated by T-DMRs at promoters of genes encoded negative regulators.

As the first step to elucidate the biological role of DNA methylation, this study examined the correlation between methylation changes and gene expression changes. Our study only included a limited number of tissues from mouse and human, and more extensive analysis is needed in future to generalize the observation. Furthermore, functional assays are also needed to substantiate our bioinformatics findings. For example, to clarify the association between DNA methylation and TF binding, TF binding data (e.g. ChIP-seq data) in corresponding cell lines and tissues are necessary to exhibit the exact TF recruiting by DNA methylation. In our published work, we demonstrated that some TFs were preferentially bound to methylated DNA [23]. We expect that some of the T-DMRs are associated with the changes of TF recruitment.

## Methods

### Identification of T-DMRs between mouse retina and brain

Methylation enrichment was performed on adult retina and brain tissue from C57BL/6 J mice (n = 3) as previously described [11]. Briefly, methylation-enriched DNA was compared to input DNA by hybridization to a custom 2.1 M NimbleGen CHARM array. In total, 2,498 T-DMRs were identified between retina and brain [21].

### Gene expression in mouse retina and brain

We performed analysis of gene expression differences between adult mouse retina (triplicates) and brain (duplicates) [12]. The gene expression data were provided in Additional file 3: Table S2. When calculating the correlation between gene expression difference (ΔE) and methylation difference (ΔM), we only considered the genes with |ΔE| > log_2_(1.25). If ΔE and ΔM had the same sign, we consider them having positive correlation and if different signs, negative correlation.

### T-DMRs and genes for human tissues

We preformed the similar T-DMRs analysis on the data from another independent study [10]. T-DMRs were identified for p < 0.001. The genes were selected if their amplitudes of gene expression difference were larger than log_2_1.5 between brain and liver, or between colon cancer and normal tissues. We chose the threshold so that the similar number of differentially expressed genes were identified for human and mouse.

### Tissue-enriched and shared DNase I hypersensitivity sites (DHSs)

Genomic DHS data for 8-week old mouse retina and brain were downloaded from the UCSC Genome Browser website: “DNaseI Hypersensitivity by Digital DNaseI from ENCODE/University of Washington” [17-19]. DHSs were identified as narrow signal peaks with false discovery rate (FDR) of 1.0% [17-19]. The DHSs present in both retina and brain were denoted shared DHSs, whereas those found only in retina or brain were denoted tissue-specific DHSs. As there were 7 replicates for mouse brain, only regions verified by 3 out of 7 replicates were defined as brain DHSs.

### Z-scores of number of genes with multiple incoherent/coherent T-DMRs

In total, 247 genes were identified with multiple T-DMRs. We kept the same ΔE for each gene and shuffled the ΔM of T-DMRs across these genes. We then determined the positive and negative correlation between ΔE and ΔM for the 247 genes. The number of genes with coherent and incoherent T-DMRs was counted in the random simulation. This process was repeated 10,000 times. To evaluate the significance of the observed number of genes with coherent and incoherent T-DMRs, we calculated the Z-score such that

$$ Z=\frac{n-\overline{S}}{std(S)} $$

where *n* is observed number of genes with incoherent/coherent T-DMRs, *S* is the set of numbers of genes with shuffled incoherent/coherent T-DMRs after 10 k times. $\overline{S}$ and *std*(*S*) are the mean value and standard deviation of *S* based on randomly shuffling results.

### Gene ontology (GO) analysis

For the tissue-enriched genes associated with T-DMRs defined above, we calculated the enrichment ratio of the occurrence of each associated GO term in the group [37], either positive or negative regulation, to that of the background genes. The background composed of 10910 genes, which are expressed in retina and/or brain, and have corresponding probes on the CHARM array. The statistical significance of the p-value was evaluated based on the hypergeometric distribution model then corrected by Bonferroni multiple-test correction. Enriched GO terms were selected with modified p-value less than 0.05.

### Motif discovery

To determine the molecular basis of DNA-methylation dependent gene regulation, we predicted DNA motifs associated with the T-DMRs. The T-DMRs were divided into two groups: negative and positive, based on the correlation of DNA methylation with gene expression. For comparison, we randomly selected 25,000 sequence segments from the genes with probes in CHARM array, which is about 10 times of number of T-DMRs. The random sequences were selected from upstream 4 kb to the end of transcription of the genes. Every random sequence has the same length of one selected T-DMR. Therefore, the random sequences have the similar length distribution to that of the T-DMRs identified. We enumerated all possible 6-mers and compared their occurrence in the T-DMRs to the random sequences. To evaluate statistical significance of the occurrence of a given 6-mer, a p-value was calculated by binominal cumulative functions [21]. False discovery rate was calculated using the Benjamini and Hochberg approach [38]. Only 6-mers with corrected p-values less than 1% (for all T-DMRs) or 5% (for T-DMRs within DHS) of FDR were selected as significant motifs. A motif was termed “dual” if a 6-mer was significantly enriched in both the negative and positive T-DMRs.

### CpG ratio of the T-DMR

CpG ratio of the T-DMR was calculated by frequency of CpG on the T-DMR divided by multiplication of frequency of C and G [5].

### Statistical models for p-value calculation

We used hypergeometric distribution to calculate p-value unless otherwise noted.

### Ethics approval for mice research

All experimental procedures were approved by the Johns Hopkins University Institutional Animal Care and Use Committee (IACUC) and were performed in accordance with guidelines established in the National Research Council’s Guide for the Care and Use of Laboratory Animals.

### Data deposition

The DNA methylation data can be downloaded at Gene Expression Omnibus (http://www.ncbi.nlm.nih.gov/geo) (GSE46683). The gene expression data is included in Additional file 3: Table S2.

## Additional files

Additional file 1: Table S1. Significantly enriched motifs were identified in all gene-regulating T-DMRs and/or those T-DMRs within DHS. The p-values were corrected by FDR. Additional file 2: Figure S1. Correlation of enrichment score of 6-mers’ occurences in all gene-regulating T-DMRs and those within DHS. The top and bottom plots are for negative regulation and positive regulation, respectively. **Figure S2.** Positive T-DMRs have higher CpG ratios than negative T-DMRs (p = 4.1 × 10^−6^, two-group t-test). **Figure S3.** Correlation of 6-mer occurrence enrichment scores in all gene-regulating. Additional file 3: Table S2. Gene expression profiles for mouse retina (triplicates) and brain (duplicates) by Affymetrix exon microarray.

## Abbreviations

CHARM: Comprehensive high-throughput array for relative methylation
C-DMR: Cancer-specific differentially methylated region
DHS: DNase I hypersensitivity site
DMR: Differentially methylated region
GO: Gene ontology
T-DMR: Tissue-specific differentially methylated region
TF: Transcription factor
TSS: Transcription start site
