# Supplementary figures and images for: Characterization of tissue-specific differential DNA methylation suggests distinct modes of positive and negative gene expression regulation
Source: BMC Genomics. 2015 Feb 5;16(1):49. doi: 10.1186/s12864-015-1271-4 (PMC4331481; doi:10.1186/s12864-015-1271-4)

**Supplemental Figure 1**

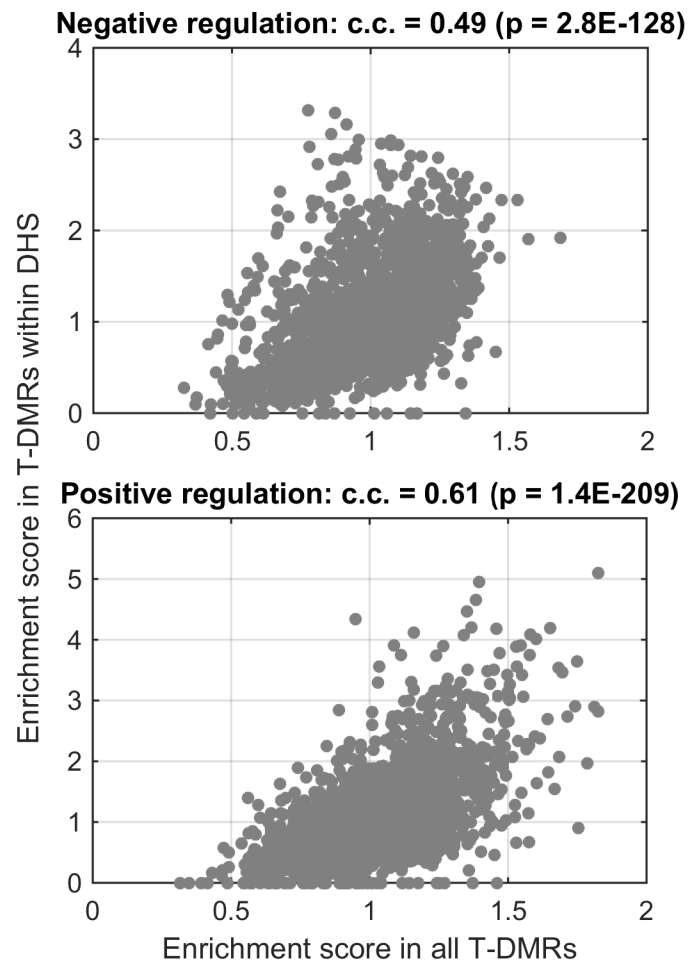

**Supplemental Figure 2**

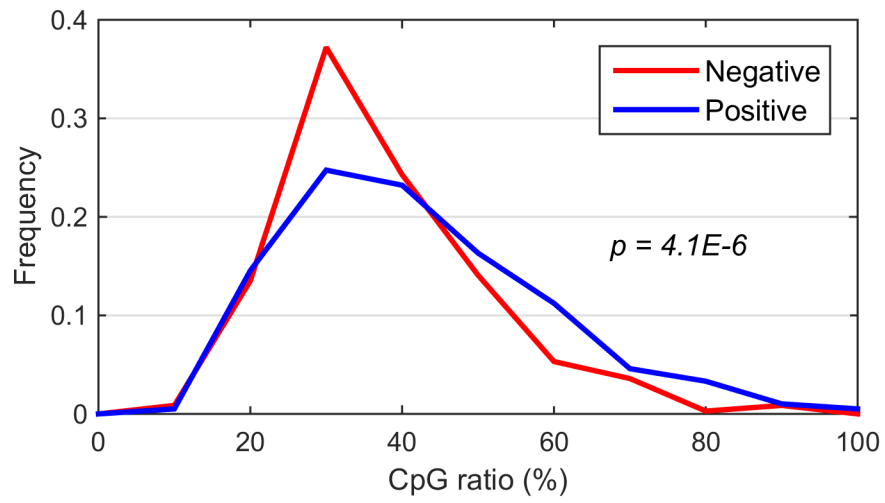

**Supplemental Figure 3**

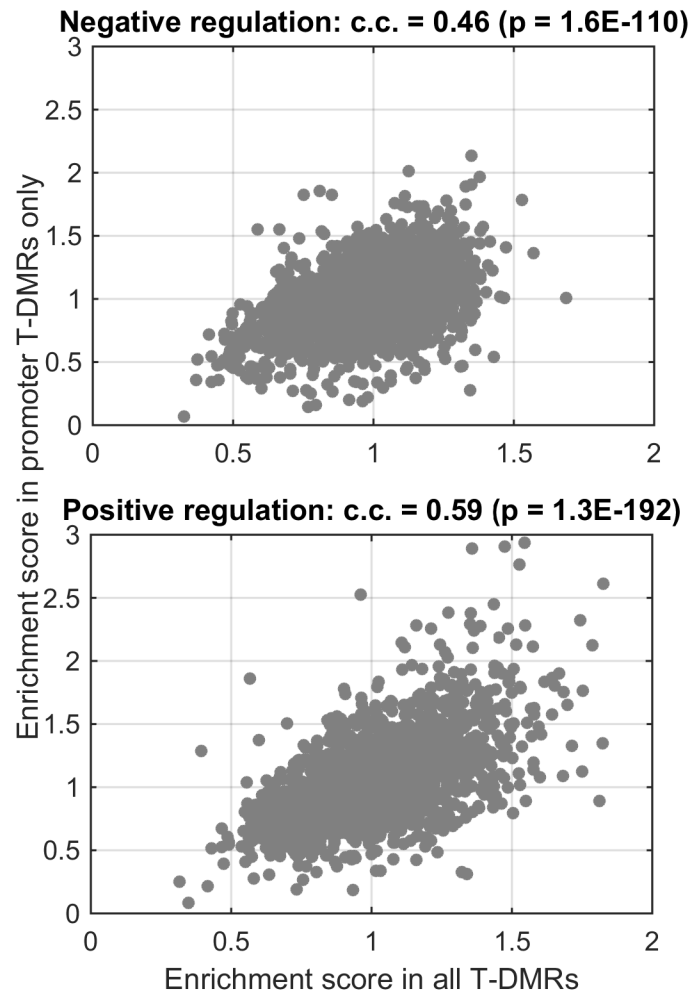

Supplement: Additional file 2: Figure S1. — Correlation of enrichment score of 6-mers’ occurences in all gene-regulating T-DMRs and those within DHS. The top and bottom plots are for negative regulation and positive regulation, respectively. Figure S2. Positive T-DMRs have higher CpG ratios than negative T-DMRs (p = 4.1 × 10^−6^, two-group t-test). Figure S3. Correlation of 6-mer occurrence enrichment scores in all gene-regulating. [file 12864_2015_1271_MOESM2_ESM.pdf]
